# A Preliminary Exploration of Landscape Preferences Based on Naturalness and Visual Openness for College Students With Different Moods

**DOI:** 10.3389/fpsyg.2021.629650

**Published:** 2021-06-03

**Authors:** Kankan Li, Yang Zhai, Long Dou, Jianjun Liu

**Affiliations:** ^1^College of Landscape Architecture and Art, Northwest Agriculture and Forestry University, Xianyang, China; ^2^Psychological Counseling Center, Northwest Agriculture and Forestry University, Xianyang, China

**Keywords:** Profile of Mood States, mood, restorative environment, environmental psychology, urban environments

## Abstract

The interaction between man and nature causes people to have different preferences for their surrounding environment, and pleasant landscapes can bring both physical and mental benefits to people. Previous studies have demonstrated the relationship between moods and landscape preferences, and this study sought to explore the landscape preferences of college students under different moods. A total of 1,034 students participated in the survey, recovering 1,022 valid questionnaires. The Profile of Mood States (POMS) scale was used to evaluate the mental status of each respondent. The study on landscape characteristics proceeded in two steps (comprising four gradients): landscape naturalness and landscape visual openness. The research results show that under natural landscape conditions, college students in a fatigued state have a greater preference for the second-gradient (higher naturalness) landscape environment; under the conditions of landscape visual openness, college students in an indignant state have a greater preference for the second-gradient (relatively private) landscapes. These findings have significance for exploring the rehabilitation function of landscape architecture and have a guiding role for future landscape design.

## Introduction

Interactions between man and nature are frequently studied through investigations of landscape perception and preferences (Appleton, 1975). People are more willing to choose the public space in line with their preferences for dynamic or static activities (Golding et al., 2018; Gagliardi and Piccinini, 2019). Therefore, landscapes need to be constantly modified according to human preferences (Schroeder, 1989; Luzar and Diagne, 1999; Erickson et al., 2002; Zheng et al., 2011), and this principle is also important for governments when formulating and implementing policies (Zheng et al., 2011). Generally, the research on landscape preference focuses on stable differences between people, such as nationality (Parsons et al., 1998), gender (Strumse, 1996), previous life experiences, and social status (van den Berg and Koole, 2006). However, many studies also focus on dynamic changing differences, such as cognitive status (Wilkie and Stavridou, 2013) or mood (van den Berg et al., 2003; Hartig and Staats, 2006; Korpela and Ylén, 2009).

Mood is defined as the way people feel at a particular time, and it is a relatively long-lasting emotional state (Zhu, 1995). Moods are more stable than feelings but are relatively less stable than personality traits (Martin and Lawson, 1998). Mood is characterized by its non-oriented dispersivity. This suggests that a desirable or undesirable mood has the ability to embed various emotional changes in an individual's mind (Zhu, 1995). College students in undesirable moods are challenged in their studies and college life (Dyson and Renk, 2006). Research shows that the environmental quality of a campus can affect students' quality of life and academic performance (Hajrasouliha, 2017). In recent years, the beneficial effects of campus environmental quality on student health have attracted the attention of a growing number of researchers (Felsten, 2009; Wu et al., 2014; Hajrasouliha, 2017). Past studies have shown that landscape preference is associated with not only human outdoor activities (Eriksson and Nordlund, 2013) and physical and mental recovery effects in the outdoor environment (Wilkie and Clouston, 2015) but also the cognition, local identity, and a psychological state of human exposure to the environment (van den Berg and Jorgensen, 2014). As a result, exploring the landscape preferences of college students with different moods is of practical significance for further research on the extent to which exposure to landscape can restore human physical and mental health. In this research, the landscape preferences of college students in different moods were explored to find commonalities and differences in their preferred spatial characteristics.

The research on landscape preference is usually based on attention recovery theory (ART) (Kaplan, 1995). According to ART, environmental aesthetics developed based on the early human selection of habitats that met these needs. This view is consistent with an evolutionary perspective (Appleton, 1975; Ulrich, 1983). Appleton (1975) was the first to explain the formation of human preferences for the environment from an evolutionary perspective. According to his theory, there is a general preference for landscapes with “prospect” and “refuge” features, as humans evolved valuing these features in environments (Mealey and Theis, 1995). An open prospect landscape offers a good view, and a landscape with refuge features could create a sense of security from unpredictable dangers. Based on Appleton's studies, Mealey and Theis (1995) argued that landscape preferences are not invariant and can be affected by moods. Nesse (1991) found that human emotional states are closely related to their landscape preferences, for example, people with positive emotions prefer open landscapes, and people with negative emotions prefer landscapes with rich elements. This study was conducted based on this logic, expecting to explore the characteristics of human landscape preferences more specifically during different mood states.

### Studying Landscape Preferences With Visualization

Visualization methods have been widely used in landscape design and the study of attributes affecting human landscape preferences (Zheng et al., 2011). For example, Tyrväinen et al. (2006) used computer-based visualization and landscape lab methods to help the public perceive their surroundings better. Ode et al. (2008) established a connection between landscape aesthetic theory and visual indicators. Numerous studies have shown that images can have restorative benefits similar to those in the real world (Berman et al., 2009; Berto et al., 2010; Kjellgren and Buhrkall, 2010), and simple visualization of the environment also has this effect (Ryan et al., 2010). Therefore, this image selection method was adopted in this research.

**Figure 1:**
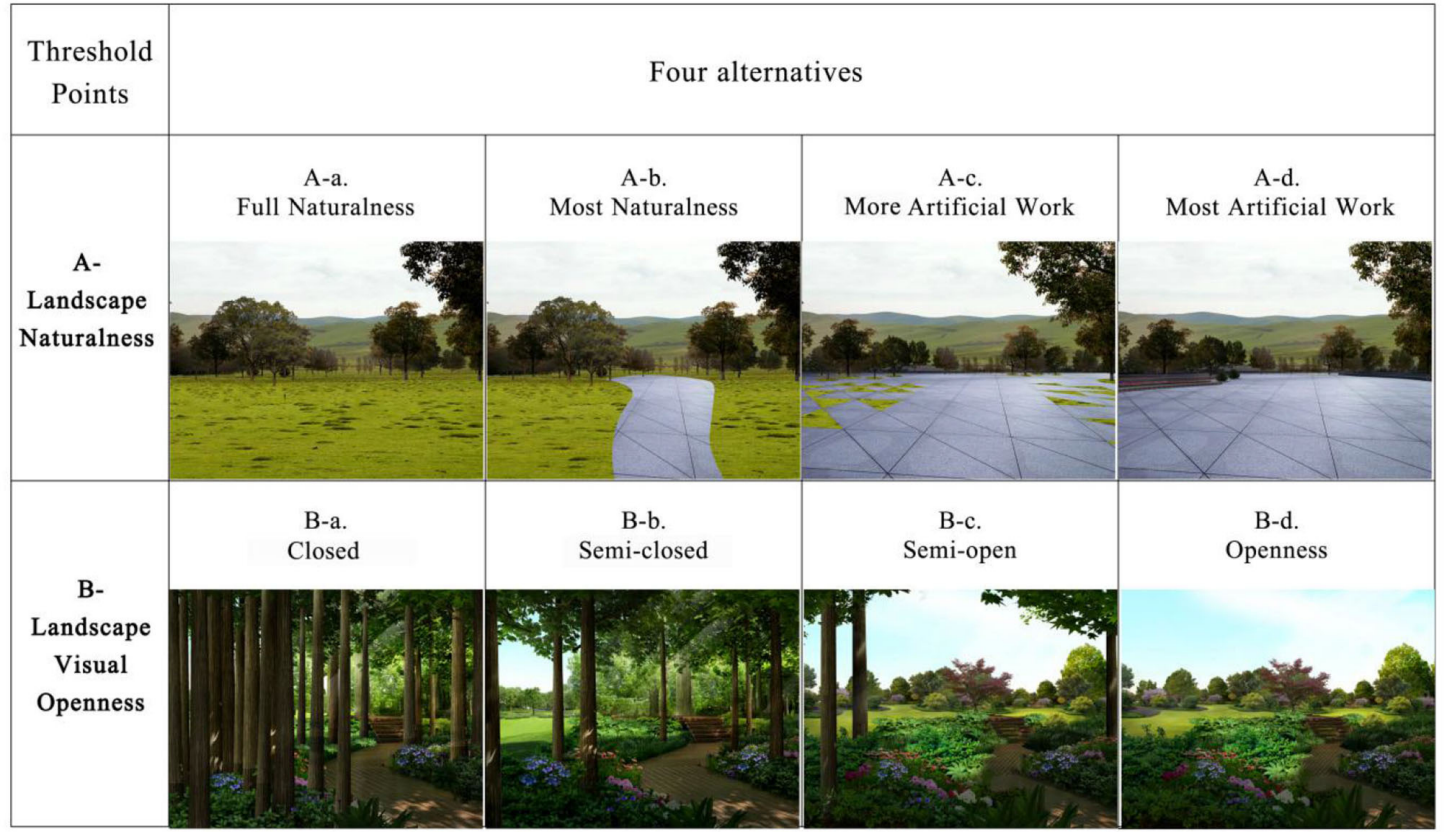
Photographs in the questionnaire survey. (1) The four alternatives (a–d) in group A were divided based on an estimate of the area of naturalness in the pictures: (A-a) is <5% of the artificial work, (A-b) is about 5–25% of the artificial traces, (A-c) is about 25%−55% of the artificial work, and (A-d) is about 55–75% of the artificial work; (2) The four alternatives (a–d) in group B were divided by the landscape's visual openness. The four degrees of a/b/c/d in group B were estimated according to the depth/high [D/H] of the site in the photos. (B-a) is D/H ≤ 1, (B-b) is 1.5 > D/H > 1, (B-c) is 2 > D/H > 1.5, and (B-d) is D/H > 2.

For visual image content, previous studies have shown that landscape preference is focused on environmental attributes (Hartig and Staats, 2006). Researchers have primarily applied the significant difference “dichotomy” comparison method to explore the positive influences of urban green land diversity on rehabilitation effects, such as comparisons between the natural environment and urban streets (Velarde and Fry, 2007; Golding et al., 2018). However, a more refined classification method can be used for groups in different mood states to positively explore the rehabilitation effects of nature (van den Berg and Jorgensen, 2014). Therefore, an analysis of landscape naturalness was incorporated into this research. In addition, the refined nature classification method proposed by van den Berg and Jorgensen (2014) was also employed in this study, which classified naturalness into four elaborate gradients (Figure 1).

Landscape components and diverse characteristics of spatial structures will compel different groups of people to express discrepant landscape preferences (Yu, 1990). Many existing landscape studies focus on the relationship between spatial privacy and functions. Moreover, the enormous influence of the visual openness of a place on a user's feelings has already been proved (Booth, 1983). Appleton argued that human preferences were derived from a sense of security that relies on landscapes characterized by “foregrounds” and “refuges” that were developed over the course of evolution; gradually, this sense of security became potentially pleasurable (Appleton, 1975; Mealey and Theis, 1995). From the perspective of landscape spatial configuration, an environment with the characteristics of “prospect” and “refuge” needs to consider whether the person exposed to the landscape has an open view or not. Additionally, a study by Windhorst and Williams (2015) observed that respondents generally acknowledge that an environment beneficial to an individual's psychological health should comprise (i) a relatively private space; (ii) a space for relaxation; (iii) a space for self-reflection; and (iv) a space with beautiful memories. Among the above-mentioned four characteristics, the first three are closely related to the landscape's spatial structure, while the last is related to personal experience. As a result, this research incorporated visual openness so as to expound the relationship between the degree of privacy of a place and the psychological feelings of users. Since Windhorst and Williams' findings on “relatively private spaces” do not specify a specific level of privacy in detail, this study attempts to analyze human preference for visual openness in different emotional states based on previous research results.

### Psychological-Affective, Mood, and Landscape Preference

Landscape preferences are defined as the extent to which people like a landscape or find it attractive (White and Gatersleben, 2011; Eriksson and Nordlund, 2013). The interactions between man and nature cause people to have different preferences for their surrounding environments, and pleasant landscapes can bring both physical and mental benefits (Kaplan and Kaplan, 1989; Kaplan et al., 1998; Ode and Fry, 2002). Many previous studies have directly or indirectly demonstrated that emotional states are closely related to landscape preferences (Nesse, 1990, 1991; Mealey and Theis, 1995; Hartig and Staats, 2006; Korpela and Ylén, 2009). A considerable number of previous studies have indicated that the beneficial effects of landscape preferences and rehabilitation landscapes on humans' physical and psychological health will be affected by a person's attention to nature, sense of fatigue, and other psychological states (Berman et al., 2009; Tyrväinen et al., 2014; van den Berg and Jorgensen, 2014; Windhorst and Williams, 2015). Although Joye and van den Berg (2011) have pointed out that landscape preference was not a direct cause of physical and mental recovery, it is undeniable that in the studies on landscape preference, there is a positive correlation between a psychological state and a healthy relationship (Hartig et al., 1997; van den Berg et al., 2003; Subiza-Pérez et al., 2019); the researchers considered that people are more likely to choose to ascend in their well-being and emotional state of active space (Kaplan et al., 1998; Feng and Chen, 2020). Although moods cannot be totally equal to emotions, moods are often used as indicators of emotion to explore the benefits of nature to human beings. Korpela and Ylén (2009) proposed that the construction of favored environments has a positive effect on human moods and cognitive recovery. Hartig and Staats (2006) targeted people with a fatigued mood to study their landscape preferences for forests and urban environments. Their results showed that participants' preferences for walking in the forest are stronger than their preferences for walking in a city; this difference increases as the respondent's fatigue level increases. As a result, this study was conducted to determine whether there was a link between mood and landscape preference.

The Profile of Mood States (POMS) scale, a common international tool to study mood status, was established by Mcnair et al. (1971) and Shahid et al. (2011). POMS is mainly used in landscape studies to determine whether exposure to landscape environments can affect specific human mood indicators. For example, being in a landscape or viewing photographs of a landscape is known to influence a detailed emotional index of moods. Park et al. (2011) and Koizumi et al. (2018) demonstrated that a well-designed city environment can mitigate emotional pressure and restore the physical and mental health of a respondent, as measured by POMS. This effect is almost equal to that of a charming natural environment. Park et al. (2011) conducted experiments in 14 forests and districts in Japan to observe how city and forest landscapes influence human physical and mental health. POMS was used as the measurement tool for mood status. A total of 168 respondents participated in the experiment. The researchers discovered that after viewing or walking through the forest, negative mood scores, such as those for tension, depression, anxiety, fatigue, and anger, decreased sharply, while positive mood scores rose dramatically. The total scores of emotional status indicated a positive effect (Park et al., 2011). These studies have proved the close relationship between moods and natural environment. In addition, these results are consistent with the view of evolution theory (Appleton, 1975; Ulrich, 1983) and ART (Kaplan, 1995). In the field of medicine and psychology, POMS data can be separated into seven mood state indicators (Koizumi et al., 2018). Based on the above findings, every sample was given seven emotional scores after the respondents completed the POMS survey. The item with the highest score was considered the mainstream mood state of the respondent. This is an innovative approach that differs from comparing the seven emotional components together, as is the case in most landscape studies. This method could make landscape preference research more targeted and is also a more informed approach. Morgan and Johnson (1978) found that the seven emotions formed a rolling line, and that athletes performed better when the highest peak of this line corresponded to vigor. The mood corresponding to the peak plays a key role in an individual's overall state. The authors referred to this method in this study because this meticulous research method could provide future reference significance for exploring the natural ability of a landscape to restore a bad state of subhealth among the population and could also provide a basis for future design practices that pay more attention to landscape functionality.

The following hypotheses were tested in this study:

H1: Respondents in positive and negative moods grouped by their Total Mood Disturbance (TMD) scores will have different preferences for landscape naturalness and landscape visual openness.
H2: Respondents in different mainstream moods will have different preferences for landscape naturalness and landscape visual openness.

## Methods

The purpose of this study was to investigate the types of landscape features most preferred by college students. Data were collected from April to June 2016, since studies have shown that certain types of scenery in some seasons may lead to negative emotions among students, such as fallen leaves in autumn and withered branches in winter. These seasonal landscape changes have been shown to contribute to depression in some people, whereas other seasonal factors such as examinations or job hunting periods could also contribute to changes in mood. The survey period in the present study was during a stage of the semester, which the authors considered unlikely to engender either positive or negative emotions among the student participants.

### Participants

The participants were simply randomly sampled. The participants were recruited through the Bulletin Board System (BBS) on campus and by forwarding the recruitment information between the students on campus. Before participating in the investigation, the students were informed of the content and purpose of the investigation; all of them volunteered to participate in the investigation and provided written consent to participate. The students were informed that they could stop filling out the questionnaire or quit at any time during the process. Questionnaires with all the answers choosing the same options are considered invalid, and questionnaires with self-evaluation (extremely excited or particularly low) within 3 days are also considered invalid. A total of 1,034 students participated in the survey, and 1,022 questionnaires were valid. Among these 1,022 participants, 43.5% were male, and 56.5% were female. The average age of the participating students was 20.1.

### Ethics

This research was conducted under the supervision of professional psychological consultants from the Psychological Consulting Centre at X University. Students participated in the survey voluntarily rather than by random selection.

### Instrument

In this study, the Simplified POMS developed based on McNair's original version by Grove and Prapavessis (1992) was used as the psychological measurement tool. The initial purpose of this tool was to evaluate experimental mood changes and emotional states. The experiments included brief mental treatments, emotional stimulation, and other similar operations. POMS is recognized as the standard tool to evaluate emotional status and has been verified as a valid experiment tool in Mainland China (Wang and Lin, 2000). In order to revise the simplified POMS Chinese norm, the researchers randomly tested 1,060 college students (including college students) in 22 provinces and cities of China, including 778 males and 282 females, and randomly selected 85 subjects from all the norm samples to test the correlation between the scores of each subscale and the corresponding questions. The results show that the reliability of Chinese POMS is between 0.62 and 0.82, with an average *r* = 0.71 (Zhu, 1995). This model has seven components: tension and anxiety (T–A), anger and hostility (A–H), depression and dejection (D), fatigue (F), vigor (V), confusion (C), and esteem-related effects (E). The average correlation coefficient of reliability is α = 0.798. This scale can be used not only to categorize respondents into seven different mood statuses but also to perform a TMD test. TMD was calculated by adding the respondents' scores for tension and anxiety, depression and dejection, anger and hostility, and fatigue and confusion, and then subtracting the respondent's vigor and esteem-related effects score. Higher TMD scores represent more obvious negative moods (Zhu, 1995). It has been shown that the Simplified POMS is a useful tool to investigate emotional status based on numerous experiments (Zhu, 1995; Wang and Lin, 2000; Deng et al., 2021).

### Questionnaire Survey

An online questionnaire was the principal survey method in this study. The Psychological Consulting Center of X University released the survey to its online student forum. Students were permitted to forward the survey to WeChat Moments (a popular social media platform in China). The online questionnaire comprised of three parts: basic information, mood state measurement, and choice of a landscape picture. The online investigation method was used to quickly collect many samples and determine whether any inherent connection existed between mood state and landscape preferences.

In the first part or basic information, there were items such as the respondents' age, gender, grade, and primary place of residence before enrollment (city, small town, or countryside).

In the second part or mood state measurement, the international universal POMS scale was used to design 40 questions that could reflect the mood state of the respondents during the preceding week. The instructions of the psychological test asked the respondents about their mood state over the past 3 days and guided them to fill in the table. The beginning of the questionnaire is a set on the three options: especially excited, particularly depressed, and normal, and to eliminate abnormal emotions caused by the deviation investigation results. The test produces eight points for each person: seven points for moods and one TMD score. All of the scores were calculated by a computer program. The participants were also told that they could receive their eight points via email after completing the questionnaire if they wished.

In the third part, picture choice questions on landscape preferences were involved. Before the participants started selecting their preferences, they had to preview the four images and then choose the scene that they liked best. After this, the participant automatically entered the second question, browsed the pictures first, and then chose their selection. Altogether, eight pictures were classified into the landscape naturalness or landscape visual openness groups. Each group had four different gradients of pictures to represent the properties of the respondent. These pictures were created by the respondent group using Photoshop CS3 software. In designing these images, the authors used one image and created four alternatives from it to ensure that each set of images involved the same scene, height, and angle. In consideration of the reality of online investigation, the size of each picture could change, along with the size of the network terminal screen, and the respondents could only see one full-sized picture at a time. Simultaneously, before selecting a picture, the respondents were given instructions to focus on the naturalness or visual openness of the picture and to imagine themselves in the place depicted in the picture (Figure 1).

## Results

### Statistics and Grouping of Respondents According to Dominant Mood Status

First, Microsoft Excel 2019 was used to make descriptive statistics of the questionnaire. The dominant mood of each respondent was selected based on the highest score in the section on moods. In this way, the respondents were categorized into one of the seven dominant moods (Table 1). Based on the TMD of the mood status on the POMS, the average TMD value of the participating respondents was 122.09. In total, 490 participants had higher scores than the average (negative mood), and 532 participants had lower scores than the average (positive mood).

**Table 1:** Seven dominant mood samples.

**Dominant mood^a^**	**T–A**	**A–H**	**D**	**F**	**V**	**C**	**E**
Subject No.^b^	109	231	66	36	559	12	9
**Gender**							
Male	42	137	21	14	223	5	3
Female	67	94	45	22	336	7	6
**Education level**							
First grade	30	75	12	11	179	5	2
Second grade	19	46	12	9	101	4	2
Third grade	26	34	15	8	86	1	2
Fourth grade	25	38	11	5	111	2	2
Above fourth grade	9	38	16	3	82	0	1
**Preschool living area**							
City	50	100	31	13	247	3	7
Small town	37	82	18	15	192	6	2
Countryside	22	49	17	8	0	3	0

a *A represents tension and anxiety, A–H, anger and hostility; D, depression and dejection; F, fatigue; V, vigor; C confusion; and E, esteem-related effects*.

b *Among these, several individual samples recorded the same scores under two mood statuses and were scored according to the discretion of the TMD*.

### Multiple Comparisons of the Differences in Landscape Features Caused by Varied Dominant Moods

#### Correlation Analysis

IBM SPSS 20.0 software was used to analyze the data. Spearman's correlation analysis was conducted to explore the relationship between landscape preferences and participants' gender, education level, preschool living area, mood, TMD, and positive/negative moods.

According to Table 2, college students' preference for naturalness is significantly related to their preschool living area, and it is not significantly related to college students' gender, education level, mood TMD, and whether their mood is positive or negative. College students' preference for visual openness of the landscape is extremely significantly correlated with their mood, TMD, and positive/negative moods, significantly correlated with their gender, and not significantly correlated with their education levels or places of residence.

**Table 2:** Correlation analysis.

	**Naturalness**		**Visual openness**	
	**Related coefficient**	**Sig. (two-tailed)**	**Related coefficient**	**Sig. (two-tailed)**
Gender	−0.032	0.303	0.069^*^	0.029
Education level	0.049	0.116	0.033	0.289
Preschool living area	−0.099^**^	0.002	−0.021	0.494
Mood	−0.022	0.473	0.088^**^	0.005
TMD	−0.035	0.257	−0.088^**^	0.005
Positive/negative moods	0.07	0.835	0.094^**^	0.003

* *P < 0.05 indicates that there is a significant difference*.

** *P < 0.01 indicates that there is a highly significant difference*.

**Figure 2:**
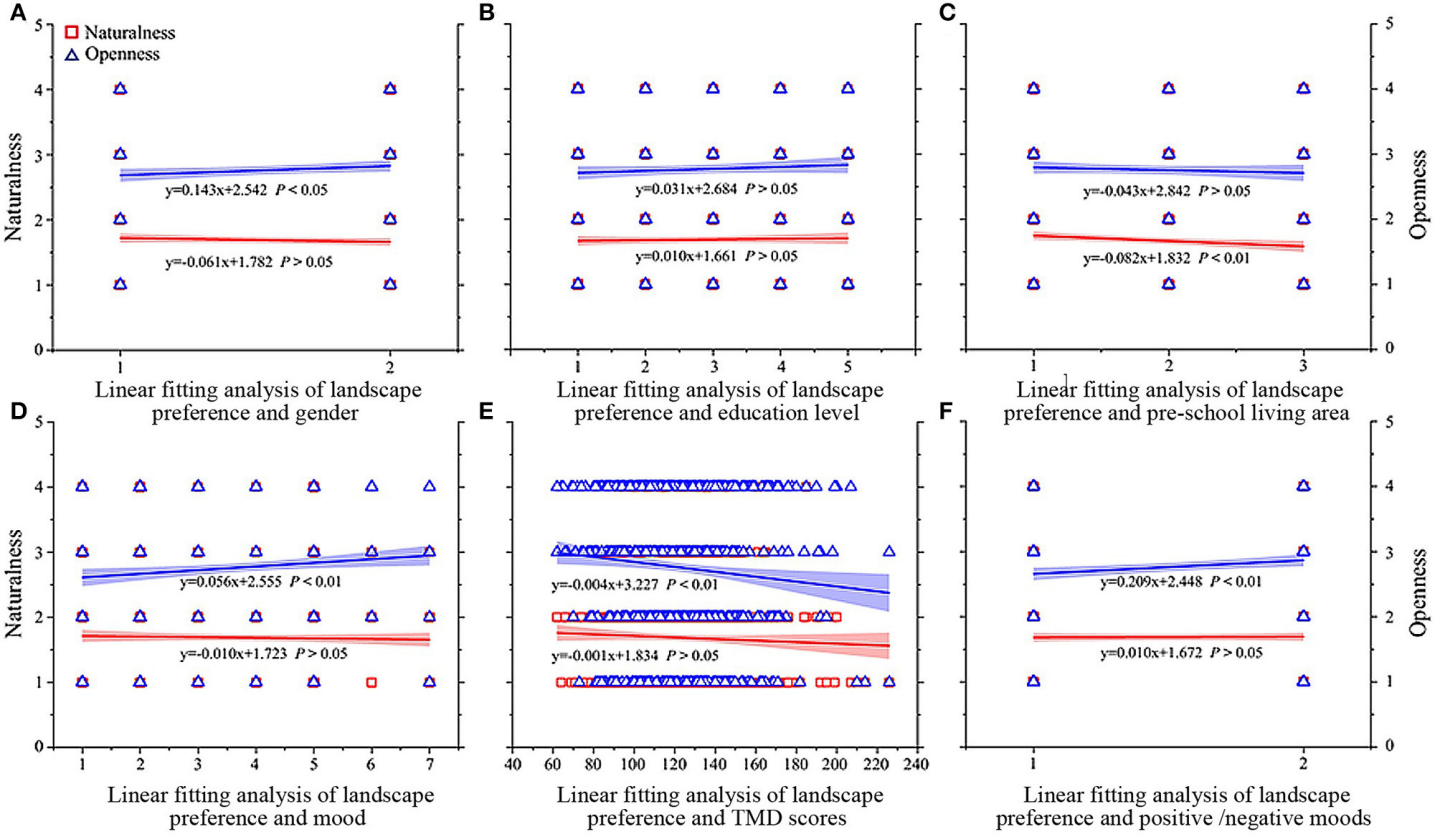
Linear fitting analysis graph. Naturalness: 1, full naturalness; 2, most naturalness; 3, most artificial work; 4, full artificial work; Visual openness:1, closed; 2, semi-closed; 3, semi-open; 4, openness; Gender: 1, male; 2, female; Education level:1, first grade; 2, second grade; 3, third grade; 4, fourth grade; 5, above fourth grade; Preschool living area:1, city; 2, small town; 3, countryside; Mood:1, T-A; 2, A-H; 3, D; 4, F; 5, V; 6, C; 7, E; Positive and negative moods: 1, negative moods; 2, positive moods.

For the second step, the authors further analyzed the fit of the sample's sociological demographic factors (gender, education level, and preschool living area), mood-related indicators, and their landscape preferences, and visualized the results as shown in Figure 2.

The research results show that the survey respondents' gender, preschool living area, mood, TMD, and positive/negative moods have a significant impact on landscape preferences (including naturalness and openness) (*P* < 0.05), while education levels have no significant impact on landscapes preference (Table 2 and Figure 2). From the perspective of the naturalness of the landscape space, the landscape preference has a significant negative correlation with the survey object's preschool living area (*P* = −0.099, *P* < 0.01; Table 2 and Figure 2C) but has no significant correlation with other factors. From the perspective of the openness of the landscape space, a significant positive correlation with gender, mood, and positive and negative (*P* = 0.069, 0.088, and 0.091; Table 2 and Figures 2A,D,F), and TMD showed a significant negative correlation (*P* = −0.088; Table 2 and Figure 2E).

#### The Difference in Preference for Landscape Naturalness

**Figure 3:**
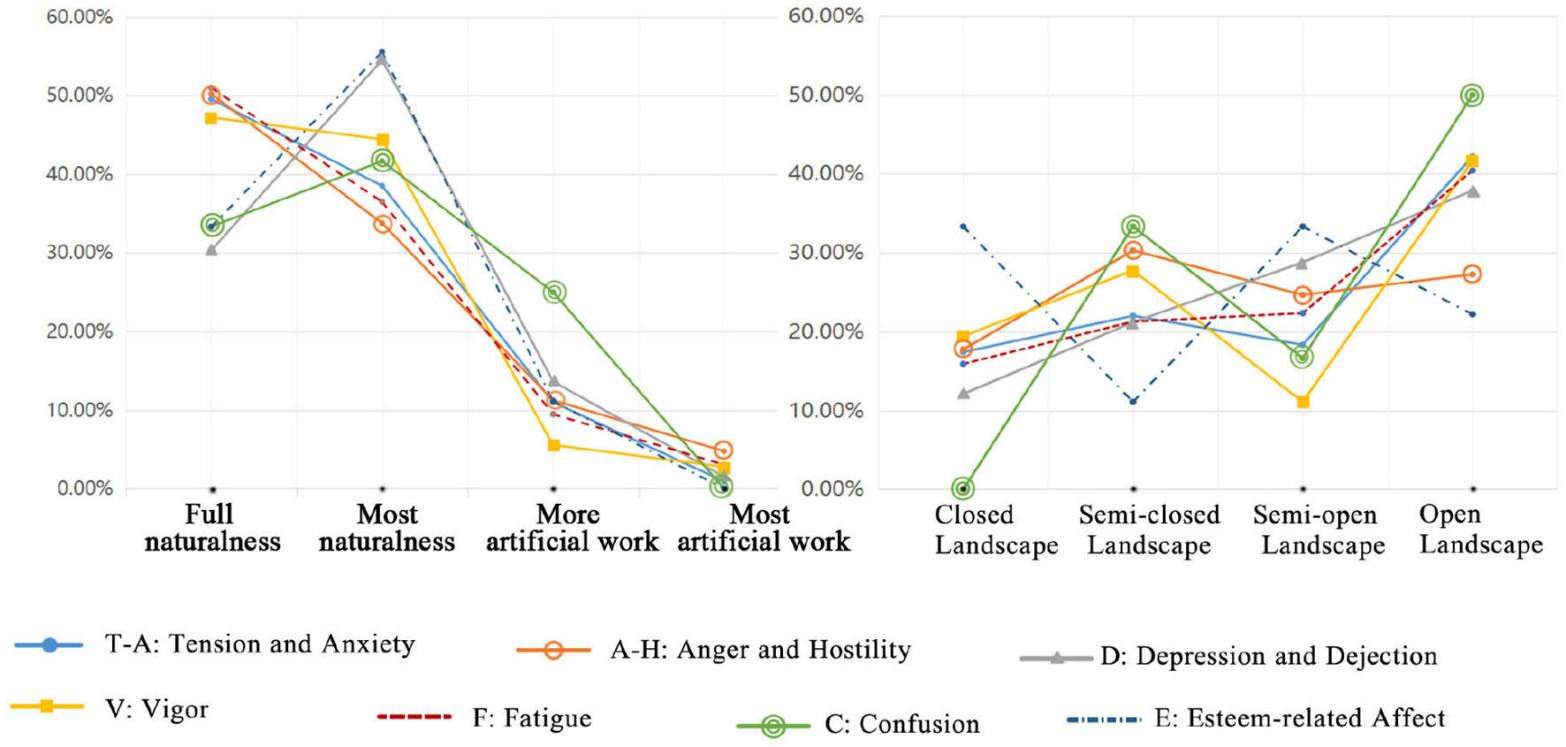
Descriptive statistics of the landscape preference selections from the perspectives of the respondents with seven different dominant moods (this illustration is based on the percentage of the selected sample).

Respondents with every mood status clearly preferred A-a and A-b rather than A-c and A-d (Figures 1, 3). Therefore, there was no difference in the preference for landscape naturalness and variation based on the respondents' mood statuses. They all preferred natural and nature-dominated landscapes.

However, as determined by the multiple comparison analyses from the perspective of landscape naturalness, the mean difference between depression and vigor was 0.218 (S.E. = 0.102, *P* = 0.032). This illustrates that there is a significant difference between the respondents experiencing dominant depression and vigor moods with the same degree of landscape naturalness. Compared with a completely natural landscape, 54.55% of the respondents in a depressive mood chose a nature-dominated landscape with a slight trace of a built environment. The preferences of the respondents in the esteem-related effects and depressive moods were similar: instead of a completely natural landscape, 55.56% of the respondents considered a nature-dominated landscape to be better (Figure 3). However, 25% of the respondents in a confused mood chose a landscape dominated by a built environment, with a slight trace of nature. The authors noted that the mood values of the respondents in a confused mood, choosing this type of photograph, were higher than those of the respondents in other moods.

#### The Difference of Preference for Landscape Visual Openness

Most of the respondents under varied mood states preferred a completely open environment (Figure 3). A small number of the respondents preferred the completely closed spaces. It was also found that an intermediate number of the respondents chose semi-closed and semi-open spaces, so there were no significant differences based on mood. Moreover, the authors discovered through multiple comparisons that the mean difference between anger and depression was −0.310 (S.E. = 0.154, *P* = 0.045). Therefore, for the same degree of landscape visual openness, there is a significant difference between the respondents with anger- and depression-dominated moods, and a very significant difference between the respondents with anger- and vigor-dominated moods. In addition, there is no significant difference between any other mood status pairs.

By recording the number of the respondents who chose each of the provided photographs, the authors found that 30% of the respondents in the anger- and hostility-dominated mood chose semi-closed spaces. This percentage is higher than that for the respondents who chose semi-open and open spaces. The respondents in a depression-dominated mood had a gradually increasing tendency to choose closed, semi-closed, semi-open, and open spaces (12.12, 21.21, 28.79, and 37.88%, respectively). The authors also found a significant difference among the respondents in an E-dominated mood compared with those with the other six moods, in terms of choosing photographs that could restore their physical and mental health: 33.33% chose closed spaces, 33.33% chose semi-open spaces, 11.11% chose semi-closed spaces, and only 22.22% chose open spaces, the last of which was preferred by all the respondents with the other six dominant moods.

#### The Consistency and Differences of the Two Perspectives for Positive and Negative Moods

**Figure 4:**
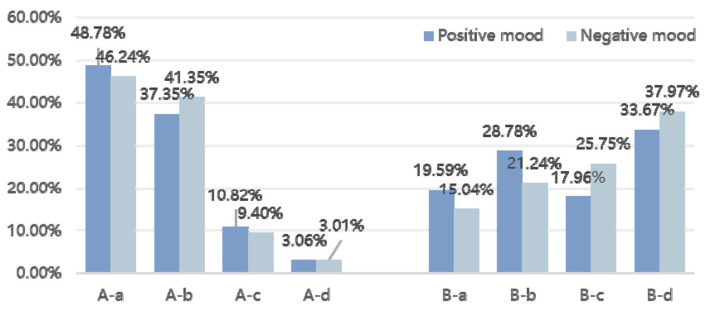
The consistency and differences of the two perspectives for positive and negative moods based on the percentage of selected samples.

As discussed above, based on the TMD of the mood status in POMS, the average TMD value of the participant samples was 122.09; 490 samples had higher-than-average values (negative moods), and 532 samples had lower-than-average values (positive moods). From the perspective of landscape naturalness, the preferences for positive and negative moods were in agreement. From the perspective of landscape visual openness, the tendency toward B-b (semi-closed) for people in negative moods was slightly higher than that of people with positive moods (Figure 4).

As shown in Table 3, when there is a difference between the TMD scores, the respondents' preferences for landscape naturalness will be significantly different.

**Table 3:** Difference between the TMD values for landscape naturalness and visual openness.

	**Sum of squares**	***df***	**Mean square**	***F***	***P***
Naturalness	5147.648	3	1715.883	2.763	0.041^*^
Visual openness	10797.698	3	3599.233	5.848	0.001^**^

* *P < 0.05 represents a significant difference*.

** *P < 0.01 represents a highly significant difference*.

According to the differences in the TMD scores, multiple comparisons were made to explore the differences in terms of landscape visual openness. As shown in Table 4, there is a significant difference in the landscape openness between B-a and B-c, B-b and B-c, and B-c and B-d. Therefore, it can be concluded that in the absence of TMD, landscape openness will influence the landscape preferences of the respondents.

**Table 4:** Multiple comparisons of the seven varied dominant moods with regard to landscape visual openness.

**(I) Preschool living area**	**(J) Preschool living area**	**Mean difference**	**Standard error**	**Significance**	**95% Confidence interval**	
					**Lower limit**	**Upper limit**
B-a	B-b	−0.69	2.496	0.782	−5.59	4.21
	B-c	8.039	2.522	0.001^**^	3.09	12.99
	B-d	1.616	2.3	0.482	−2.9	6.13
B-b	B-c	8.728	2.284	0^**^	4.25	13.21
	B-d	2.306	2.037	0.258	−1.69	6.3

** *P < 0.01 indicates that there is a highly significant difference*.

## Discussion

This research was conducted to enhance understanding of the relationship between the environment and the physical and mental health of people (Zheng, 2009). It is demonstrated that college students in varied moods all prefer natural landscapes and open view landscapes, a result that is consistent with previous research (Appleton, 1975; Kaplan and Kaplan, 1989; Berman et al., 2009, 2012; Bowler et al., 2010). In addition, it was also found that the landscape preferences of the college students were related to their long-term residence factors, which is also consistent with the identity research results of Clayton (2003) and Wilkie and Stavridou (2013). The landscape preferences of people are closely related to their moods because an individual's landscape preferences are primarily based on their need for directed attention restoration (Joye and van den Berg, 2011). Previous studies have shown that compared with urban environments, exposure to natural environment offers greater improvement of attention restoration for people with positive emotions, negative emotions, and fatigued mood (Berman et al., 2009, 2012; Bowler et al., 2010). However, the result of landscape preference is not the only evidence of landscape resilience, and there have been conflicting studies in this area. In some cases, scenarios that are considered highly restorative for people are not necessarily highly recommended settings (Peron et al., 2002). In addition, there is no evidence that the actual recovery of the environment can predict human-environmental preferences (Han, 2010). However, it is undeniable that environmental preferences are precursors to judgments of environmentally directed attention recovery potential (Wilkie and Stavridou, 2013; Shen et al., 2019).

Furthermore, this study specifically shows that there are significant differences in the degree of naturalness of the preferred landscape among college students with different moods. There were some inconsistencies in this pattern, as the respondents did not consider a completely natural landscape to be the only environment that would be beneficial to their physical and mental health. For instance, instead of a natural landscape, most respondents in a fatigued mood preferred a nature-dominated landscape with a small amount of built environment. These findings are also consistent with those of Kaplan and Kaplan (1989), who demonstrated the human preference for complex landscapes.

Several studies have focused on the influence of nature or built environments on the restoration of human physical and mental health over time (Jackson, 2003; Wilkie and Stavridou, 2013). Wilkie and Stavridou (2013) categorized environmental types into nature, urban green spaces, and urban street landscapes in a study on the influence of environmental types in cities on physical and mental health restoration among humans, and the potential for perceptive restoration. They demonstrated that urban green spaces and natural landscapes have an identical effect on restoring human physical and mental health (Wilkie and Stavridou, 2013). Others have stated that a well-designed city environment can mitigate pressure and restore human physical and mental health (Cattell et al., 2008; Abdulkarim and Nasar, 2014; Bratmana et al., 2015). This effect is equivalent to that of an attractive natural environment (Koizumi et al., 2018). However, the authors discovered from this research that only a small percentage of the respondents prefer a built or urban environment. This difference is probably due to the negative impression urban landscapes may leave on people (Kaplan and Kaplan, 1989; Ulrich et al., 1991).

On the other hand, there is a close interrelationship between visual openness and satisfaction with an open space (Francis et al., 2012). Shach-Pinsly (2010) showed that people prefer places with wide views, such as parks or adjacent buildings in a high-density city. Hidetoshi et al. (1995) stated that the higher the visual openness of a public space, the higher the quality of the environment, and the higher the satisfaction of residents in the living space. The above findings are consistent with the results of this study, showing that students with different moods prefer to choose a scene with wide visual openness. This wide visual openness does not entail complete emptiness, but there are certain lines of sight restrictions. Therefore, landscape architecture practices should include diverse spaces in the future that are characterized by variable landscape visual openness designs, thereby providing people in different moods with convenient access to restorative places.

### Limitations

Previous studies have demonstrated that if a photograph of a natural landscape retains the complete information of the scene, reactions toward the real landscape and the photograph should be consistent (Ulrich et al., 1991; Chang et al., 2008). However, in addition to sight, the senses of smell, hearing, taste, and touch are all used to gain a holistic understanding of the environment (Li, 2011). Since it is difficult to organize and inspect actual sites, visual models should be constructed in future experiments, thereby maximizing the validity of scene restoration and reduce the variation caused by uncontrolled simultaneous variables.

## Conclusions

It has been demonstrated that college students with fatigue-dominated moods prefer a nature-dominated landscape with a small amount of built environment, compared with those in other moods. College students with anger-dominated moods prefer a semi-closed space. Only a small percentage of the students with esteem-related moods preferred open spaces; however, open spaces were the most commonly selected spaces by the students experiencing other moods. Most respondents prefer a private or small-scale space that can occupy two to four people, regardless of mood. The students with depressed and confused moods preferred private spaces. In this study, the seven moods were divided into positive and negative categories. The people with both positive and negative moods consistently preferred landscape naturalness. However, with regard to visual landscape openness, the tendency toward semi-closed spaces for people with negative moods was slightly higher than for people with positive moods. With regard to landscape sociality, the percentage of the respondents in negative moods that preferred private spaces was significantly higher than for the respondents with positive moods.

It is believed that exploring the landscape preferences of college students under different moods has practical significance in determining to what extent human physical and mental health can be restored through exposure to specific landscapes. This study has shown that human landscape preferences are associated with their moods. The campus is not only the main place of study and living for a student but also an important space where students can use natural methods to cope with and relieve pressure. As a result, the campus landscape should have more natural elements to increase students' contact with nature. In addition, based on these findings, it is necessary to design diverse spaces that include variable landscape visual openness in the future landscape architecture practices, thereby providing people in various mood states with convenient access to restorative places.

## Data Availability Statement

The raw data supporting the conclusions of this article will be made available by the authors, without undue reservation.

## Ethics Statement

The studies involving human participants were reviewed and approved by Northwest Agriculture & Forestry University Ethics Committee. The patients/participants provided their written informed consent to participate in this study. Written informed consent was obtained from the individual(s) for the publication of any potentially identifiable images or data included in this article.

## Author Contributions

KL and JL: conceptualization. KL: methodology, formal analysis, investigation, writing—review and editing, and project administration. YZ: software. LD: resources. YZ: data curation. KL and YZ: writing—original draft preparation. JL: funding acquisition. All authors contributed to the article and approved the submitted version.

## Conflict of Interest

The authors declare that the research was conducted in the absence of any commercial or financial relationships that could be construed as a potential conflict of interest.

## References

[B1] Abdulkarim D. Nasar J. L. (2014). Are livable elements also restorative? J. Environ. Psychol. 38, 29[entx]x02013[/entx]38. 10.1016/j.jenvp.2013.12.003

[B2] Appleton J. (1975). The Experience of Landscape. London: John Wiley and Sons. 66[entx]x02013[/entx]67.

[B3] Berman M. G. Jonides J. Kaplan S. (2009). The cognitive benefits of interacting with nature. Psychol. Sci. 19, 1207[entx]x02013[/entx]1212. 10.1111/j.1467-9280.2008.02225.x 19121124

[B4] Berman M. G. Kross E. Krpan K. M. Askren M. K. Burson A. Deldin P. J.. (2012). Interacting with nature improves cognition and affect for individuals with depression. J. Affect. Disord. 140, 300[entx]x02013[/entx]305. 10.1016/j.jad.2012.03.012 22464936 PMC3393816

[B5] Berto R. Baroni M. R. Zainaghi A. Bettella S. (2010). An exploratory study of the effect of high and low fascination environments on attentional fatigue. J. Environ. Psychol. 30, 494[entx]x02013[/entx]500. 10.1016/j.jenvp.2009.12.002

[B6] Booth N. K. (1983). Basic Elements of Landscape Architectural Design. New York, NY: Elsevier.

[B7] Bowler D. E. Buyung-Ali L. M. Knight T. M. Pullin A. S. (2010). A systematic review of evidence for the added benefits to health of exposure to natural environments. BMC Public Health 10:456. 10.1186/1471-2458-10-456 20684754 PMC2924288

[B8] Bratmana G. N. Daily G. C. Levyc B. J. Gross J. J. (2015). The benefits of nature experience: improved affect and cognition. Landsc. Urban Plan. 138, 41[entx]x02013[/entx]50. 10.1016/j.landurbplan.2015.02.005

[B9] Cattell V. Dines N. Gesler W. Curtis S. (2008). Mingling, observing, and lingering: everyday public spaces and their implications for well-being and social relations. Health Place 14, 544[entx]x02013[/entx]561. 10.1016/j.healthplace.2007.10.007 18083621

[B10] Chang C. Y. Hammitt W. E. Chen P. K. Machnik L. Su W. C. (2008). Psychophysiological responses and restorative values of natural environments in Taiwan. Landsc. Urban Plan. 85, 79[entx]x02013[/entx]84. 10.1016/j.landurbplan.2007.09.010

[B11] Clayton S. (2003). [entx]x0201C[/entx]Environmental identity: a conceptual and an operational definition,[entx]x0201D[/entx] in Identity and the Natural Environment: The Psychological Significance of Nature, eds Clayton S. Opotow S. (MIT Press), 45[entx]x02013[/entx]65.

[B12] Deng X. Guo X. Wu Y. J. Chen M. (2021). Perceived environmental dynamism promotes entrepreneurial team member[entx]x00027[/entx]s innovation: explanations based on the uncertainty reduction theory. Int. J. Environ. Res. Public Health 18:2033. 10.3390/ijerph18042033 33669732 PMC7921965

[B13] Dyson R. Renk K. (2006). Freshmen adaptation to university life: depressive symptoms, stress, and coping. J. Clin. Psychol. 62, 1231[entx]x02013[/entx]1244. 10.1002/jclp.20295 16810671

[B14] Erickson D. L. Ryan R. L. Young R. D. (2002). Woodlots in the rural landscape: landowner motivations and management attitudes in a michigan (usa) case study. Landsc. Urban Plan. 58, 101[entx]x02013[/entx]112. 10.1016/S0169-2046(01)00213-4

[B15] Eriksson L. Nordlund A. (2013). How is setting preference related to intention to engage in forest recreation activities? Urban For. Urban Green. 12, 481[entx]x02013[/entx]489. 10.1016/j.ufug.2013.07.004

[B16] Felsten G. (2009). Where to take a study break on the college campus: an attention restoration theory perspective. J. Environ. Psychol. 29, 160[entx]x02013[/entx]167. 10.1016/j.jenvp.2008.11.006

[B17] Feng B. Chen M. (2020). The impact of entrepreneurial passion on psychology and behavior of entrepreneurs. Front. Psychol. 11:1733. 10.3389/fpsyg.2020.01733 32793066 PMC7385187

[B18] Francis J. Wood L. J. Knuiman M. Giles-Corti B. (2012). Quality or quantity? Exploring the relationship between public open space attributes and mental health in Perth, Western Australia. Soc. Sci. Med. 74, 1570[entx]x02013[/entx]1577. 10.1016/j.socscimed.2012.01.032 22464220

[B19] Gagliardi C. Piccinini F. (2019). The use of nature [entx]x02013[/entx] based activities for the well-being of older people: an integrative literature review. Archiv. Gerontol. Geriatr. 83, 315[entx]x02013[/entx]327. 10.1016/j.archger.2019.05.012 31128876

[B20] Golding S. Gatersleben B. Cropley M. (2018). An experimental exploration of the effects of exposure to images of nature on rumination. Int. J. Environ. Res. Public Health 15:300. 10.3390/ijerph15020300 29425140 PMC5858369

[B21] Grove J. R. Prapavessis H. (1992). Preliminary evidence for the reliability and validity of an abbreviated profile of mood states. Int. J. Sport Psychol. 23, 93[entx]x02013[/entx]109. 10.1007/BF00636229

[B22] Hajrasouliha A. (2017). Campus score: measuring university campus qualities. Landsc. Urban Plan. 158, 166[entx]x02013[/entx]176. 10.1016/j.landurbplan.2016.10.007

[B23] Han K. T. (2010). An exploration of relationships among the responses to natural scenes. Environ. Behav. 42, 243[entx]x02013[/entx]270. 10.1177/0013916509333875

[B24] Hartig T. Bk A. Garvill J. Olsson T. Grling T. (1997). Environmental influences on psychological restoration. Scand. J. Psychol. 37, 378[entx]x02013[/entx]393. 10.1111/j.1467-9450.1996.tb00670.x 8931393

[B25] Hartig T. Staats H. (2006). The need for psychological restoration as a determinant of environmental preferences. J. Environ. Psychol. 26, 215[entx]x02013[/entx]226. 10.1016/j.jenvp.2006.07.007

[B26] Hidetoshi W. Akio K. Takashi N. (1995). A study on the relation of resident[entx]x00027[/entx]s evaluation on waterfront area and living environment. Nihon kenchiku Gakkai Keikakukei ronbun hokoku shu 2, 199[entx]x02013[/entx]206. 10.3130/aija.60.199_2

[B27] Jackson R. J. (2003). The impact of the built environment on health: an emerging field. Am. J. Public Health 93, 1382[entx]x02013[/entx]1384. 10.2105/AJPH.93.9.1382 12948946 PMC1447976

[B28] Joye Y. van den Berg A. (2011). Is love for green in our genes? A critical analysis of evolutionary assumptions in restorative environments research. Urban For. Urban Green. 10, 261[entx]x02013[/entx]268. 10.1016/j.ufug.2011.07.004

[B29] Kaplan R. Kaplan S. (1989). The Experience of Nature: A Psychological Perspective. Cambridge, UK: Cambridge University Press. 10.1037/030621

[B30] Kaplan R. Kaplan S. Ryan R. L. (1998). With People in Mind: Design and Management of Everyday Nature. Washington, DC: Island Press. 10.3368/lj.18.1.99

[B31] Kaplan S. (1995). The restorative benefits of nature: toward an integrative framework. J. Environ. Psychol. 15, 169[entx]x02013[/entx]182. 10.1016/0272-4944(95)90001-2

[B32] Kjellgren A. Buhrkall H. (2010). A comparison of the restorative effect of a natural environment with that of a simulated natural environment. J. Environ. Psychol. 30, 464[entx]x02013[/entx]472. 10.1016/j.jenvp.2010.01.011

[B33] Koizumi K. Tayama J. Ishioka T. Nakamura-Thomas H. Hamaguchi T. (2018). Anxiety, fatigue, and attentional bias toward threat in patients with hematopoietic tumors. PLoS ONE 13:e0192056. 10.1371/journal.pone.0192056 29401504 PMC5798784

[B34] Korpela K.M. Yl[entx]x000E9[/entx]n M.P. (2009). Effectiveness of favorite-place prescriptions: a field experiment. Am. J. Prevent. Med. 36, 435[entx]x02013[/entx]438. 10.1016/j.amepre.2009.01.022 19269127

[B35] Li S. H. (2011). Horticultural Therapy. Beijing: Chinese Forestry Press.

[B36] Luzar E. J. Diagne A. (1999). Participation in the next generation of agriculture conservation programs: the role of environmental attitudes. J. Socio Econ. 8, 335[entx]x02013[/entx]349. 10.1016/S1053-5357(99)00021-9

[B37] Martin B. Lawson R. (1998). Mood and framing effects in advertising. Austr. Mark. J. 6, 35[entx]x02013[/entx]50. 10.1016/S1441-3582(98)70238-1

[B38] Mcnair D. Lorr M. Droppleman L. (1971). Manual for the Profile of Mood States. San Diego, CA: Educational and Industrial Testing Service.

[B39] Mealey L. Theis P. (1995). The relationship between mood and preferences among natural landscapes: an evolutionary perspective. Ethol. Sociobiol. 16, 247[entx]x02013[/entx]256. 10.1016/0162-3095(95)00035-J

[B40] Morgan W. P. Johnson R. W. (1978). Personality characteristics of successful and unsuccessful oarsmen. Int. J. Sport Psychol. 9, 119[entx]x02013[/entx]133.

[B41] Nesse R. M. (1990). Evolutionary explanations of emotions. Hum. Nat. 1, 261[entx]x02013[/entx]289. 10.1007/BF02733986 24222085

[B42] Nesse R. M. (1991). What is mood for. Psycholoquy 2:9.

[B43] Ode [entx]x000C5[/entx]. K. Fry G. A. (2002). Visual aspects in urban woodland management. Urban For. Urban Green. 1, 15[entx]x02013[/entx]24. 10.1078/1618-8667-00003

[B44] Ode A. Tveit M. S. Fry G. (2008). Capturing landscape visual character using indicators: touching base with landscape aesthetic theory. Landsc. Res. 33, 89[entx]x02013[/entx]117. 10.1080/01426390701773854

[B45] Park B. J. Furuya K. Kasetani T. Takayama N. Kagawa T. Miyazaki Y. (2011). Relationship between psychological responses and physical environments in forest settings. Landsc. Urban Plan. 102, 24[entx]x02013[/entx]32. 10.1016/j.landurbplan.2011.03.005

[B46] Parsons R. Tassinary L. G. Ulrich R. S. Hebl M. R. Grossman-Alexander M. (1998). The view from the road: implications for stress recovery and immunization. J. Environ. Psychol. 18, 113[entx]x02013[/entx]140. 10.1006/jevp.1998.0086

[B47] Peron E. Berto R. Purcell T. (2002). Restorativeness, preference, and the perceived naturalness of places. Medio Ambiente y Comporamiento Humano 3, 19[entx]x02013[/entx]34.

[B48] Ryan R. M. Weinstein N. Bernstein J. Brown K. W. Mistretta L. Gagn[entx]x000E9[/entx] M. (2010). Vitalizing effects of being outdoors and in nature. J. Environ. Psychol. 30, 159[entx]x02013[/entx]168. 10.1016/j.jenvp.2009.10.009

[B49] Schroeder H. W. (1989). [entx]x0201C[/entx]Environment, behavior, and design research on urban forests,[entx]x0201D[/entx] in Advance in Environment, Behavior, and Design, Vol. 2, eds Zube E. H. Moore G. T. (Boston, MA: Springer), 87[entx]x02013[/entx]117. 10.1007/978-1-4613-0717-4_4

[B50] Shach-Pinsly D. (2010). Visual openness and visual exposure analysis models used as evaluation tools during the urban design development process. J. Urbanism Int. Res. Placemak. Urban Sustain. 3, 161[entx]x02013[/entx]184. 10.1080/17549175.2010.502002

[B51] Shahid A. Wilkinson K. Marcu S. Shapiro C. M. (2011). Profile of Mood States (POMS). New York, NY: Springer. 10.1007/978-1-4419-9893-4_68

[B52] Shen C.-W. Min C. Wang C.-C. (2019). Analyzing the trend of O_2_O commerce by bilingual text mining on social media. Comput. Hum. Behav. 101, 474[entx]x02013[/entx]483. 10.1016/j.chb.2018.09.031

[B53] Strumse E. (1996). Demographic differences in the visual preferences for agrarian landscapes in Western Norway. J. Environ. Psychol. 14, 17[entx]x02013[/entx]31. 10.1016/S0272-4944(05)80219-1

[B54] Subiza-P[entx]x000E9[/entx]rez M. Hauru K. Korpela K. Haapala A. Lehvvirta S. (2019). Perceived environmental aesthetic qualities scale (peaqs) [entx]x02013[/entx] a self-report tool for the evaluation of green-blue spaces. Urban For. Urban Green. 43:126383. 10.1016/j.ufug.2019.126383

[B55] Tyrv[entx]x000E4[/entx]inen L. Gustavsson R. Konijnendijk C. Ode [entx]x000C5[/entx]. (2006). Visualization and landscape laboratories in planning, design and management of urban woodlands. Forest Policy Econ. 8, 811[entx]x02013[/entx]823. 10.1016/j.forpol.2004.12.005

[B56] Tyrv[entx]x000E4[/entx]inen L. Ojala A. Korpela K. Lanki T. Tsunetsugu Y. Kagawa T. (2014). The influence of urban green environments on stress relief measures: a field experiment. J. Environ. Psychol. 38, 1[entx]x02013[/entx]9. 10.1016/j.jenvp.2013.12.005

[B57] Ulrich R. S. (1983). Aesthetic and affective response to natural environment. Behav. Nat. Environ. 6, 85[entx]x02013[/entx]125. 10.1007/978-1-4613-3539-9_4

[B58] Ulrich R. S. Simons R. F. Losito B. D. Fiorito E. Zelson M. (1991). Stress recovery during exposure to natural and urban environments. J. Environ. Psychol. 11, 201[entx]x02013[/entx]230. 10.1016/S0272-4944(05)80184-7

[B59] van den Berg A. Jorgensen A. (2014). Evaluating restoration in urban green spaces: does setting type make a difference? Landsc. Urban Plan. 127, 173[entx]x02013[/entx]181. 10.1016/j.landurbplan.2014.04.012

[B60] van den Berg A. Koole S. L. (2006). New wilderness in the netherlands: an investigation of visual preferences for nature development landscapes. Landsc. Urban Plan. 78, 362[entx]x02013[/entx]372. 10.1016/j.landurbplan.2005.11.006

[B61] van den Berg A. E. Koole S. L. van der Wulp N. Y. (2003). Environmental preference and restoration: (how) are they related? J. Environ. Psychol. 23, 135[entx]x02013[/entx]146. 10.1016/S0272-4944(02)00111-132964236

[B62] Velarde M. D. Fry G. (2007). Health effects of viewing landscapes [entx]x02013[/entx] landscape types in environmental psychology. Urban For. Urban Green. 6, 199[entx]x02013[/entx]212. 10.1016/j.ufug.2007.07.001

[B63] Wang J. Lin W. (2000). POMS for use in China. Acta Psychol. Sin. 32, 110[entx]x02013[/entx]114.

[B64] White E. V. Gatersleben B. (2011). Greenery on UK Residential Buildings: does it affect preferences and perceptions of beauty? J. Environ. Psychol. 31, 89[entx]x02013[/entx]98. 10.1016/j.jenvp.2010.11.002

[B65] Wilkie S. Clouston L. (2015). Environment preference and environment type congruence: effects on perceived restoration potential and restoration outcomes. Urban For. Urban Green. 14, 368[entx]x02013[/entx]376. 10.1016/j.ufug.2015.03.002

[B66] Wilkie S. Stavridou A. (2013). Influence of environmental preference and environment type congruence on judgments of restoration potential. Urban For. Urban Green. 12, 163[entx]x02013[/entx]170. 10.1016/j.ufug.2013.01.004

[B67] Windhorst E. Williams A. (2015). [entx]x0201C[/entx]It[entx]x00027[/entx]s like a different world[entx]x0201D[/entx]: natural places, post-secondary students, and mental health. Health Place 34, 241[entx]x02013[/entx]250. 10.1016/j.healthplace.2015.06.002 26093082

[B68] Wu C. D. McNeely E. Cede[entx]x000F1[/entx]o-Laurent J. G. Pan W. C. Adamkiewicz G. Dominici F.. (2014). Linking student performance in Massachusetts elementary schools with the [entx]x0201C[/entx]greenness[entx]x0201D[/entx] of school surroundings using remote sensing. PLoS ONE 9:e108548. 10.1371/journal.pone.0108548 25310542 PMC4195655

[B69] Yu K. (1990). The Plant Configuration of the Evolution of the Spatial Structure Research and Its Aesthetic History. Beijing: The Nation[entx]x00027[/entx]s Academic Press.

[B70] Zheng B. (2009). Accounting for preferences and attitudes to urban trees and residential landscapes (M.A. Thesis), Alabama: Auburn University, Forest Economics and Policy Department. Available online at: https://etd.auburn.edu//handle/10415/1650

[B71] Zheng B. Zhang Y. Chen J. (2011). Preference to home landscape: wildness or neatness? Landsc. Urban Plan. 99, 1[entx]x02013[/entx]8. 10.1016/j.landurbplan.2010.08.006

[B72] Zhu B. (1995). Brief introduction of POMS scale and its model for China. J. Tianjin Instit. Phys. Educ. 10, 35[entx]x02013[/entx]37.

